# A consideration of resistance and tolerance for ruminant nematode infections

**DOI:** 10.3389/fgene.2012.00168

**Published:** 2012-12-14

**Authors:** Stephen C. Bishop

**Affiliations:** Genetics and Genomics, Royal (Dick) School of Veterinary Studies, The Roslin Institute, University of EdinburghEdinburgh, UK

**Keywords:** resilience, sheep, worms, animal genetics, epidemiology

## Abstract

Debates on the relative merits of resistance (the ability of the host to control the parasite lifecycle) and tolerance (the net impact of infection on host performance) are often lively and unhindered by data or evidence. Resistance generally shows continuous, heritable variation but data are sparser for tolerance, the utility of which will depend upon the disease prevalence. Prevalence is a function of group mean resistance and infection pressure, which itself is influenced by mean resistance. Tolerance will have most value for endemic diseases with a high prevalence but will be of little value for low prevalence diseases. The conditionality of tolerance on infection status, and hence resistance, makes it difficult to estimate independently of resistance. Tolerance is potentially tractable for nematode infections, as the prevalence of infection is ca. 100% in animals grazing infected pasture, and infection level can be quantified by faecal egg count (FEC). Whilst individual animal phenotypes for tolerance are difficult to estimate, breeding values are estimable if related animals graze pastures of different contamination levels. Selection for resistance, i.e., FEC, provides both direct and indirect benefits from ever decreased pasture contamination and hence decreased infectious challenge. Modeling and experimental studies have shown that such reductions in pasture contamination may lead to substantially increased performance. It is proposed that selection goals addressing nematode infections should include both resistance and performance under challenging conditions. However, there may be benefits from exploiting large datasets in which sires are used across cohorts differing in infection level, to further explore tolerance. This may help to customise breeding objectives, with tolerance given greater weight in heavily parasitized environments.

## Introduction

This paper aims to consider the relative definitions of resistance and tolerance, as applied to host genetic resistance to disease in livestock, determine the situations when resistance and tolerance are useful breeding goals, and apply the concepts discussed to nematode infections in ruminants. Currently there is considerable debate amongst livestock geneticists on the relative utility of resistance and tolerance when considering the term “disease resistance”; such debates are often unhindered by data or evidence and are even unhindered by a consistent logical thread in the argument. Curiously a parallel debate on the merits of tolerance and resistance has been conducted within the ecological and immunological communities (e.g., Råberg et al., 2007, 2009). However, there has been little cross fertilization between these different groups of researchers. The debate on the relative merits of resistance and tolerance is particularly apposite now, as disease resistance is becoming an ever more ubiquitous goal in many breeding programs and is invariably nominated by breeders as a high priority trait. Further, with the ready availability of DNA from populations of animals that have faced epidemic challenges, genomic selection (albeit with low precision) is now becoming an option for diseases that hitherto would have been difficult to incorporate into breeding programs.

From consideration of literature on disease resistance (from a livestock viewpoint) it is apparent that different authors have different interpretations of the term “resistance”. For example, common usage is to define resistance in terms of susceptibility to infection *per se*, i.e., liability to becoming infected when faced with an infectious challenge of a parasite or pathogen, with animals that are less susceptible being more resistant. However, this definition does not hold for nematode infections, where faecal egg count (FEC) is often used as the indicator of relative resistance and FEC may be thought of summarizing the net outcome of the host–parasite interaction. The issue of trait definition for resistance has even been avoided on occasions. For example Boddicker et al. (2012), in a study aiming to find QTL for resistance to porcine reproductive and respiratory syndrome (PRRS), simply described their trait (viraemia following infection) as a measure of host response to infection.

The trait definition problem can be clarified to some extent by generalizing the definitions to encapsulate the trait biology, as outlined by Bishop and Stear (2003). Defining infection as the colonization of a host animal by a parasite (or pathogen) and disease as the side effects of infection, these authors then defined resistance as the ability of the individual host to control or influence the parasite (pathogen) lifecycle, and tolerance as the net impact of infection on the performance of host animal, i.e., the disease side-effects. These definitions are consistent with those used elsewhere in this Special Topic. Definitions as broad as this allow the concepts of resistance and tolerance to be applied to any disease, and to be applied equally to any aspect of the host–parasite (pathogen) interaction or any outcome of infection. Full definitions of the terms used in this paper to describe impacts of infection on individual hosts and in populations are shown in Box 1, along with a diagrammatic representation of these terms.

Box 1 **Definitions used in paper.**

**Resistance**

The ability of the host animal to exert control over the parasite or pathogen lifecycle. *Measurements which indicate level of parasite burden are often considered to be indicators of resistance. Such traits include faecal egg count, viraemia, or bacterial load in animals infected with nematodes, viruses, or bacteria, respectively.*

**Tolerance**

The net impact on performance of a given level of infection. *Measurement of tolerance is logistically difficult as discussed here and by Doeschl-Wilson et al. (2012a,b)*

**Resilience**

The productivity of an animal in the face of infection. *Resilience is often measured simply as performance in an infected environment, however indirect measurements such as treatment requirements are sometimes used as a proxy.*

**Prevalence**

The proportion of the host population that is infected or diseased at a specific point in time.

**Incidence**

The number of new cases that arise in a population over a specified time period. *Incidence is a rate parameter and is often incorrectly confused with prevalence*.

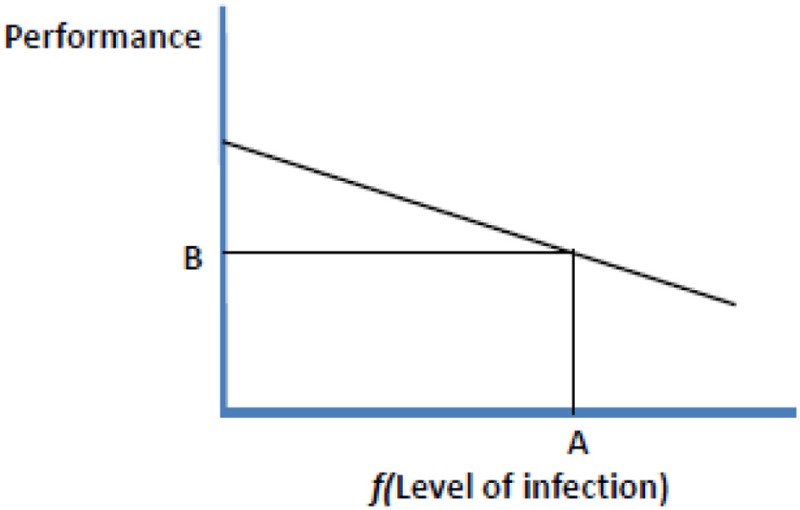

The figure shows a schematic representation of performance and level of infection, or some function that linearises the relationship between level of infection and performance. The regression slope represents **Tolerance**, point A indicates **Resistance** and point B represents **Resilience**.

This review article considers the wider implications of resistance and tolerance, when applied to any infectious disease, with a particular focus on nematode infections in ruminants. It is assumed that for most diseases host–parasite interactions are complex and under partial genetic control (e.g., Davies et al., 2009). Further, it is assumed that the complexity of the host–parasite interactions leads to variation in resistance being polygenic in most (but not all) cases.

## Defining tolerance in an epidemiological context

The relative merits of resistance and tolerance as breeding goals depend upon the nature and epidemiology of the disease. Self-evidently, tolerance is only expressed when animals are infected, therefore the value of tolerance depends, amongst other factors, on the epidemiology of the disease. Two factors come into play, the interpretation (and hence utility) of tolerance and the estimation of tolerance.

### Interpretation and utility of tolerance

Tolerance can only be expressed once an animal has become infected. Thus, with a given prevalence (*p*) of infection (see Box 1), a proportion *p* of the population will express tolerance and 1-*p* will not. As *p* approaches unity, tolerance becomes a useful concept, however as *p* falls to levels such that a large proportion of animals are not infected, its utility becomes questionable.

Let us assume initially that tolerance is estimable at the individual animal level, and hence that infection status of individual animals is known. The information available for tolerance within a population will depend upon many factors, including those which determine the true prevalence of infection, and those which determine the observed prevalence, conditional on the true prevalence. The latter is largely a function of the accuracy of the diagnostic tests, i.e., the specificity (*S*_p_, this being the probability that a truly *uninfected* individual is classified by the diagnostic test as *uninfected)* and sensitivity (*S*_e_, this being the probability that a truly *infected* individual is classified by the diagnostic test as *infected)*. As shown by Bishop and Woolliams (2010), the regression of observed on true prevalence is *p*′ = (1 − *S*_*p*_) + (*S*_*p*_ + *S*_*e*_ − 1)*p*, hence imperfect diagnostic tests will reduce heritabilities by a factor (*S*_*p*_ + *S*_*e*_ − 1)^2^, and estimated SNP effects by a factor (*S*_*p*_ + *S*_*e*_ − 1).

Factors affecting true prevalence are more complex, depending upon the force of infection (Anderson and May, 1991). Thus, prevalence is influenced both directly and indirectly (through the infectivity of infected animals) by the mean resistance of the population. Therefore, the utility of tolerance is partly dependent on the mean resistance of the population, with tolerance becoming more valuable as mean resistance is decreased. However, at the individual animal level, and assuming that resistance to infection is heritable, it is the least resistant (or most susceptible) animals that are the most likely to become infected, and hence the most likely to yield information on tolerance. Thus, the expression of tolerance is conditional upon the individual animal's resistance to infection.

When applying these concepts to deriving breeding goals, the first point to note is that tolerance is most useful when *p* approaches unity. As soon as *p* drops substantially below unity, then resistance must also be included in the breeding goal, else there is a risk that selection pressure is targeted toward the least resistant animals in the population and away from those that are more valuable from a disease control perspective, *viz*. the more resistant animals.

A further point is that tolerance should not be considered as a breeding goal in situations where control of transmission of infection is paramount. Most obviously this applies to zoonotic infections, i.e., infections harbored by animals that cause disease in humans, but it applies also to situations where other populations surrounding our target flock/herd are notably susceptible to infection.

### Estimation of tolerance

Following the definitions used by Simms (2000) and adapted for the impact of infectious diseases on animal performance by Kause (2011), we may define tolerance for the *i*th animal as the regression slope (*b*_*i*_) in the relationship: *Y*_*i*_ = *Y*_0*i*_ + *b*_*i*_*f(Ii)*, where *Y*_*i*_ is the observed performance, *Y*_0*i*_ is performance when the animal is uninfected, *Ii* is the level of infection (pathogen/parasite burden) of the animal and *f*(*x*) is some function which makes the relationship between pathogen burden and decline in performance approximately linear. This is shown diagrammatically in Box 1.

Two features of this relationship are important. Firstly, at the population level the (genetic) covariance of tolerance and performance under non-infected conditions is important [i.e., Cov(*Y*_0_, *b*)]; as one would not want decreased performance under non-infectious conditions to be an unintended consequence of selection for improved tolerance. Secondly, tolerance is likely to be difficult to measure at the individual animal level in many circumstances, as measurements of performance at two or more different levels of infection will be required, on the same animal. This concept has been explored in detail by Doeschl-Wilson et al. (2012b, this volume), and these authors propose some novel analytical solutions. However, it is difficult to envisage how individual animal tolerance could be estimated in traits expressed over a short duration or only once (such as survival, longevity, growth rate over defined time periods, or carcass characteristics). On the other hand, for traits expressed repeatedly by adult animals (such as reproductive performance, lactation traits, or fiber production) measurement may be feasible.

Simply measuring productivity of animals under disease challenging conditions is seen as a desirable breeding goal in many circumstances and by many breeders, however this does not equate to tolerance. This trait is a composite of productivity under uninfected conditions, resistance (which affects pathogen/parasite level) and tolerance, further complicated by possible covariances between these traits. Also, provided that there is genetic variation in resistance, then this trait will show genotype by environment interactions as the force of infection changes.

### Parasite/pathogen coevolution risks

As reviewed by Råberg et al. (2009), it has been long argued that tolerance places less selective pressure on the pathogen to evolve than resistance, hence tolerance should be a more sustainable selection criterion. Further, in an evolutionary context, selective pressure on mutations enhancing tolerance (where the “performance” trait is fitness) will tend to fixation (Roy and Kirchner, 2000), whereas selective pressure on mutations for resistance will decrease as the allele frequency increases due to the genetic equivalent of herd immunity. These arguments are backed empirically by the observation that livestock living in areas with high infectious disease challenge generally tend to be tolerant of infection rather than resistant, a prime example being trypanotolerance.

These arguments are, however, rather simplistic and may require modification. Firstly, these arguments ignore a third factor in the arms race, *viz*. parasite virulence. When all three traits are put together, expected outcomes over co-evolutionary time periods are complex and depend on assumed relationships amongst the traits (Carval and Ferriere, 2010). However the problem can be simplified by acknowledging that full co-evolutionary models are not necessary, as in the livestock context genetic changes in host animals are controlled and, in most cases, likely to be relatively small. To my knowledge there are no robust theoretical considerations of pressures placed on pathogen evolution through selection for resistance or tolerance, however analogous studies have been done for parasite evolution risks arising from vaccines with different modes of action (Gandon et al., 2001). Considering anti-malarial vaccines, and under the assumptions of their model, vaccines affecting susceptibility to infection, infectivity, and tolerance had somewhat similar predicted effects on parasite virulence evolution, whereas those affecting parasite proliferation led the parasite to evolve toward markedly greater virulence. In summary, it is likely that some aspects of resistance place greater selection pressure on the pathogen to evolve than tolerance, however this argument should be stated in shades of gray rather than black and white.

### Synopsis

Tolerance may be a useful concept for some diseases, depending upon the epidemiological context. Further, it provides an advantage over resistance insofar as tolerance of infection, in many circumstances, may place less selective pressure on the pathogen than resistance. However, there are a number of caveats to beware of when considering tolerance as a breeding goal. First, it is not desirable for zoonotic infections. Secondly, its value depends upon the prevalence of infection, decreasing as prevalence decreases. Thirdly, as prevalence decreases, there is a risk that selection intensity can only be achieved for the least resistant animals, implying that tolerance should never be decoupled from resistance within a breeding goal. Finally, the range of traits for which tolerance can be easily or unambiguously estimated at an individual animal level may be limited to those repeatedly expressed by adult animals over the course of their lifetime.

One class of diseases that meets most of the criteria necessary for tolerance to be a feasible selection goal is nematode infections, particularly in ruminants. This paper will now focus on the application of tolerance to nematode infections.

## Application to nematode infections

### Introduction to the issue

Gastrointestinal nematode parasite infections, particularly of ruminants, are probably the class of disease with the greatest impact upon animal health and productivity, particularly in developing countries where they have a large impact on the livelihoods of livestock keepers (Perry et al., 2002). Furthermore, they also represent an important disease issue in developed countries, especially in the sheep and goat sector. However, nematodes represent a threat to any extensively kept livestock species.

Much work has been done quantifying genetic variation for many aspects of the host response to infection in small ruminants [see summary by Bishop and Morris (2007)], and heritable variation is nearly always observed. Further, numerous studies have shown that selection for resistance is possible and effective in both sheep (Woolaston and Piper, 1996; Woolaston and Windon, 2001; Morris et al., 2005; Karlsson and Greeff, 2006; Kemper et al., 2010a) and goats (Vagenas et al., 2002), and indeed it is now widely implemented in several countries, notably New Zealand and Australia. Such selection should reduce costs of parasitism and increase the shelf-life of anthelmintics in the face of widespread evolution of anthelmintic resistance in nematodes (Waller, 1997; Jackson and Coop, 2000).

Despite the apparent success in breeding sheep for resistance to nematodes, considerable debate still exists on the best phenotype to use for selection, i.e., should it be resistance, tolerance or resilience? NB resilience may be thought of as the productivity of an animal in the face of infection (see Box 1). I have previously discussed this topic (Bishop, 2012), however here I consider it further. It should firstly be pointed out that nematode infections do lend themselves, in principle, to breeding for tolerance or some related trait as the prevalence of infection is invariably close to 100% and nematode infections are not zoonotic.

### Definition and consequences of selectable traits

The indicator trait most conveniently used to describe resistance to nematode infections is FEC. This is a composite trait, being the product of (female) worm burden and worm fecundity. Because it is invariably heavily right-skewed, it is often log-transformed prior to analysis, hence we might expect the heritability of FEC to be close to the average of the heritabilities for worm burden and worm fecundity. These are difficult traits to measure, however when all three traits have been measured this expectation does hold true (Stear et al., 1997; Davies et al., 2005). Whilst FEC may be a good indicator of worm burden for nematode species such as *Haemonchus contortus* and *Trichostrongylus colubriformis*, for *Teladorsagia circumcincta* this relationship breaks down at high worm burdens due to density-dependent constraints on worm fecundity (Bishop and Stear, 2000).

More generally, many measurements have been used to quantify variation in impacts of nematode infections on host animals, and these have previously (Bishop, 2012) been classified as follows: (1) measures of resistance: FEC, worm burden, worm size, and fecundity; (2) measures of immune responses: e.g., eosinophilia, antibodies such as IgA, IgG, IgM; (3) measures of impact of infection: e.g., anaemia (as measured by packed cell volume (PCV) or eyelid color), pepsinogen or fructosamine concentrations; (4) various direct and indirect measures of resilience: including growth rate, anthelmintic requirements (“the age at which a first post-weaning anthelmintic treatment is required to maintain acceptable growth in lambs grazing nematode-contaminated pasture,” Morris et al., 2010) and anaemia following *H. contortus* infection (Baker et al., 2003). Clearly categories (3) and (4) overlap in their definitions.

Selecting for increased resistance, i.e., decreased FEC, has an additional advantage of leading to both direct and indirect (epidemiological) benefits resulting from ever decreased pasture contamination and hence decreased infectious challenge. Several modeling studies have shown that whilst the direct impacts of selection for reduced FEC on performance traits depend on the genetic correlations between traits (e.g., Vagenas et al., 2007; Doeschl-Wilson et al., 2008), the reductions in pasture contamination (from reduced FEC) potentially lead to substantially increased performance (Bishop and Stear, 1997, 1999; Laurenson et al., 2012). Various experimental studies now support these theoretical predictions of epidemiological benefits arising from populations of animals excreting fewer eggs (Gruner et al., 2002; Leathwick et al., 2002; Williams et al., 2010).

A potential down side of selection for increased resistance is the possibility of evolution of the nematode population, analogous to the evolution of anthelmintic resistance in response to indiscriminate use of anthelmintics. This topic has been considered in detail by Kemper et al. (2009, 2010b), Kemper (2010) and summarized by Bishop (2012). Briefly, experimental evidence has failed to show that nematodes adapt differentially to resistant and susceptible hosts, at least as far as the experimental system had power to detect such effects (Kemper et al., 2009). Secondly, modeling studies have suggested that the advantages of resistant hosts in terms of reduced FEC should be maintained for many host generations. These results are partially due to the highly polygenic nature of variation in resistance (Kemper et al., 2011), and the expected slow rates of parasite evolution are in stark contrast to those expected for anthelmintic resistance.

With the exception of FEC and growth rate under parasitized conditions, which is self-evidently a selection criterion in nearly all sheep breeding programs, of the other selection criteria mooted above, it appears only to be selection for decreased treatment requirements (an indirect indicator of resilience) that has been implemented. Indeed, Morris et al. (2010) found that long-term selection for decreased treatment requirements was effective, albeit complex to implement, leading to decreased breech soiling, and increased growth rate whilst not altering resistance. Although the other immunological or metabolic traits are invariably heritable and genetically correlated with nematode resistance traits, I am not aware of long-term selection performed on such traits in ruminants. However, it may be wise to exercise caution before advocating selection on indicator traits before their time- and challenge-dependent properties are known. For example, Davies (2006) estimated genetic correlations of indicator traits such as IgA or eosinophil concentrations with FEC or worm fecundity across different ages. Not only did she find that the correlations changed over time, but they often changed sign between times when lambs presumably had immature immune responses (e.g., immediately post-weaning at 3 months of age) and when they had more mature immune responses (e.g., 6 months). Therefore, the age and exposure history of animals must be clearly defined before selecting on traits that change with increasing exposure.

### Consideration of tolerance

To date, the discussion has avoided the concept of tolerance, i.e., the decline in performance as infection level increases. At the breed level, genetic differences in impacts of infection in sheep, presumed to be tolerance, have been clearly and elegantly demonstrated in the comparison of Red Maasai and Dorper sheep, in environments differing in level of challenge (Baker et al., 2004). In fact, this was interpreted as a genotype by environment interaction, as described above in section “Estimation of Tolerance.” The Red Maasai breed is considered to be relatively resistant and is termed resilient, on account of its PCV levels following exposure to *H. contortus* (Baker et al., 2003). The same authors observed the Dorper breed to be considerably more susceptible and less resilient to *H. contortus*. Productivity of these two breeds varies considerably according to environment, with Red Maasai sheep being more efficient than Dorper sheep in a high challenge environment, whereas in a low challenge semi-arid environment there were negligible breed differences in productive efficiency (Baker et al., 2004). Even after accounting for differences in resistance, this equates to the Red Maasai breed being more tolerant of infection than the Dorper breed.

In principle, measurement of tolerance at the breed or group level is relatively straightforward for nematode infections, as the measurements required to estimate tolerance (i.e., performance and FEC at different levels of challenge) are readily available [see Kause (2011) and Doeschl-Wilson et al. (2012a), this volume]. At a level down from the group level, there may also be possibilities to assess tolerance at the sire family level. Large datasets that are now becoming available as a result of industry- or breed-wide breeding programs may provide the data to allow this, provided that nematode resistance traits (i.e., FEC) have been measured alongside performance traits. Using data where sires have been evaluated across years and across farms differing in infection level may permit the estimation of tolerance at the sire level. If this succeeds, it may enable customization of breeding objectives by environment, with (sire) tolerance given greater weight in environments that provide greater parasite challenges.

Measurement of tolerance at the individual animal is, as described above, considerably more difficult. Although the major impact of nematode parasitism is on growing lambs, it will almost certainly not be possible to assess performance on the same lamb at different levels of challenge, as challenge level and exposure-dependent acquisition of immunity will be confounded. However, in principle, individual animal tolerance can be assessed in traits expressed by adult animals. The adult ewe is generally only affected by nematode infections during the peri-parturient period, when the impacts of late gestation and early lactation lead to a temporary waning of immunity (Taylor, 1935). During this period, ewe “productivity” may be defined as milk production, which is reflected in the growth rate of her lambs. Genetic correlations between ewe FEC and the growth rate of her lambs during this period have been reported by Bishop and Stear (2001); these were positive suggesting a nutrient partitioning or resource allocation effect, i.e., ewes preferentially allocating resources to lactation instead of immunity tended to have lambs which grew faster simultaneously with a higher FEC (and vice versa). Whilst this suggests an impact of nematode infections on performance it does not directly give information on tolerance. However, datasets such as this, with repeated observations across years on both the infection trait (FEC) and the performance trait (lamb growth), do potentially allow estimates to be made of individual animal tolerance, regressing performance on FEC. Once estimated, this will allow exploration of the genetic properties of individual animal tolerance.

### A role for genomics

This Review so far has been mainly concerned about trait definition, i.e., the phenotypic side of genetic improvement. However, genomics may have an added-value role to play in the optimal selection for resistance/tolerance in relation to nematode infections. Genomic selection, based on concepts outlined by Haley and Visscher (1998) and Meuwissen et al. (2001) is now well-established in the dairy cattle sector. In principle, it could also be applied to the small ruminant sector although this would require a marked change in available genomic tools as small ruminants do not have the advantages seen in the dairy sector of high animal value, small effective population size, and highly accurate phenotypes (i.e., daughter trait averages) to calibrate the predictions. Further, published studies of genomic prediction of nematode resistance suggest only moderate accuracy with currently available SNP arrays (Kemper et al., 2011). However, under the assumption that more powerful genomic tools come available, the concept is worth pursuing.

The key advantage of genomic selection is that, once calibrated, it can reduce the requirement for intensive ongoing trait recording. For a trait as inherently difficult to measure as nematode tolerance, this could be advantageous. As described above, and also by Doeschl-Wilson et al. (2012a,b) tolerance is readily assessed at the group or sire family level. A trait defined at the sire family level is analogous to sex-limited traits seen in dairy cattle, such as milk production, where the EBV is estimated from progeny performance. In these circumstances genomic predictions are readily made from de-regressed estimated breeding values. Similarly in sheep, sires with progeny in high and low nematode challenge environments enable, in principle, EBVs for tolerance to be estimated for the sire, and hence genomic predictions of tolerance for next-generation animals to be made using SNP arrays. Therefore, genomics may allow prediction of individual animal tolerance to be made in situations where individual animal phenotypes are difficult to obtain.

As described above, this is an “in principle” use of genomics to help address tolerance of nematode infections in sheep. Making this work in practice would require large datasets on performance and infection levels for genetically related animals in different environments, and probably cheaper yet more powerful genomic tools than available at the time of writing.

## Conclusions

In conclusion, whilst tolerance is an appealing concept, and one that is much discussed when considering disease resistance, and also debated in ecological and evolutionary discussions, it is actually a difficult trait to use in practical situations. In particular, the complexity of measuring individual animal tolerance makes it difficult to implement into breeding programs, although novel analytical solutions to this problem are proposed in this volume (Doeschl-Wilson et al., 2012b). In many cases, geneticists believe they are measuring tolerance when in actual fact they are looking at a composite trait combining tolerance and resistance. Further, the utility of tolerance as a breeding goal depends on the epidemiology of the disease, as it is only useful when infection is prevalent.

Conceptually, the utility of tolerance in a breeding goal, assuming that it can be measured, depends on many factors, including animal genotypes for resistance, for tolerance, for productivity under situations of no challenge and the covariance amongst these traits. Further, because of the dependence of tolerance on the prevalence of infection, factors which influence prevalence also become important. This includes population mean resistance, especially the infectivity component of resistance, as this will influence the force of infection faced by the whole population.

In principle tolerance is applicable to nematode infections, as these infections usually lead to a prevalence approaching unity and nematode infections are not zoonotic. However, even in this situation tolerance is hard to estimate: it may be estimated at the breed or sire family level, but rarely can it be estimated at the individual animal level. An exception may be for nematode infections in lactating animals, because in this case data from separate lactations on the same animal may be considered as independent expressions of the same trait, with different infection levels in different years.

Whilst it may not be possible to obtain unbiased estimates of tolerance for most traits, various pragmatic solutions may capture the information necessary to design effective breeding programs. For example, by measuring resistance (FEC) and performance in a parasitized environment (sometimes referred to as resilience, see Box 1), sufficient information is available to improve both performance and resistance in that environment, with improvements in resistance leading to further indirect benefits via decreased pasture contamination. Accounting for the tolerance component of environmental sensitivity (hence genotype by environment interactions) would require information from farms varying in degree of nematode challenge, with these farms linked by usage of common sires. Such information will already be available in many structured breeding programs, enabling estimation of genotype by environment interactions, and determination of whether breeding goals customized by (parasite) environment are necessary. Therefore, in practice it may be possible to capture the benefits of tolerance to nematode infections for livestock, without necessarily having to obtain unbiased estimates of this trait.

### Conflict of interest statement

The author declares that the research was conducted in the absence of any commercial or financial relationships that could be construed as a potential conflict of interest.
